# *In silico* analysis reveals the co-existence of CRISPR-Cas type I-F1 and type I-F2 systems and its association with restricted phage invasion in *Acinetobacter baumannii*

**DOI:** 10.3389/fmicb.2022.909886

**Published:** 2022-08-17

**Authors:** Gulshan Yadav, Ruchi Singh

**Affiliations:** ^1^Indian Council of Medical Research (ICMR)—National Institute of Pathology, Safdarjung Hospital Campus, New Delhi, India; ^2^Manipal Academy of Higher Education (MAHE), Manipal, Karnataka, India

**Keywords:** *Acinetobacter baumannii*, CRISPR-Cas, co-existence, type I-F1, type I-F2

## Abstract

**Introduction:**

*Acinetobacter baumannii*, an opportunistic pathogen, rapidly acquires antibiotic resistance, thus compelling researchers to develop alternative treatments at utmost priority. Phage-based therapies are of appreciable benefit; however, CRISPR-Cas systems are a major constraint in this approach. Hence for effective implementation and a promising future of phage-based therapies, a multifaceted understanding of the CRISPR-Cas systems is necessary.

**Methods:**

This study investigated 4,977 RefSeq genomes of *A. baumannii* from the NCBI database to comprehend the distribution and association of CRISPR-Cas systems with genomic determinants.

**Results:**

Approximately 13.84% (*n* = 689/4,977) isolates were found to carry the CRSIPR-Cas system, and a small fraction of isolates, 1.49% (*n* = 74/4,977), exhibited degenerated CRISPR-Cas systems. Of these CRISPR-Cas positive (+) isolates, 67.48% (465/689) isolates harbored type I-F1, 28.59% (197/689) had type I-F2, and 3.7% (26/689) had co-existence of both type I-F1 and type I-F2 systems. Co-existing type I-F1 and type I-F2 systems are located distantly (∼1.733 Mb). We found a strong association of CRISPR-Cas systems within STs for type I-F1 and type I-F2, whereas the type I-F1 + F2 was not confined to any particular ST. Isolates with type I-F1 + F2 exhibited a significantly high number of mean spacers (*n* = 164.58 ± 46.41) per isolate as compared to isolates with type I-F2 (*n* = 82.87 ± 36.14) and type I-F1 (*n* = 54.51 ± 26.27) with majority targeting the phages. Isolates with type I-F1 (*p* < 0.0001) and type I-F2 (*p* < 0.0115) displayed significantly larger genome sizes than type I-F1 + F2. A significantly reduced number of integrated phages in isolates with co-existence of type I-F1 + F2 compared with other counterparts was observed (*p* = 0.0041). In addition, the isolates carrying type I-F1 + F2 did not exhibit reduced resistance and virulence genes compared to CRISPR-Cas(–) and CRISPR-Cas (+) type I-F1 and type I-F2, except for *bap, abaI*, and *abaR*.

**Conclusion:**

Our observation suggests that the co-existence of type I-F1 and F2 is more effective in constraining the horizontal gene transfer and phage invasion in *A. baumannii* than the isolates exhibiting only type I-F1 and only type I-F2 systems.

## Introduction

*Acinetobacter baumannii*, an opportunistic pathogen, is of great clinical relevance and is associated with hospital-acquired infections in immunocompromised patients (Munoz-Price and Weinstein, 2008). It rapidly develops resistance against all classes of antibiotics. Multidrug-resistant, extensively drug-resistant, and pan-drug-resistant strains of *A. baumannii* have now been prominently reported worldwide (Zarrilli et al., 2009; Tal-Jasper et al., 2016; Joshi et al., 2017; Bassetti et al., 2018; Leungtongkam et al., 2018) and obligated the World Health Organization to classify this pathogen as a critical priority pathogen for developing novel antibiotics (World Health Organization, 2017). In addition to novel antibiotics, alternative treatment strategies are also being explored (Baptista et al., 2018; Merker et al., 2020; Pires et al., 2020; Kumar et al., 2021; Micoli et al., 2021).

Clustered regularly interspaced short palindromic repeats (CRISPR) and CRISPR-associated (Cas) genes provide immunity against phages and other foreign genetic elements through the incorporation of spacers (Pourcel et al., 2005; Barrangou et al., 2007; Brouns et al., 2008). Approximately 36–42% of completely sequenced bacteria possess CRISPR-Cas systems (Makarova et al., 2020; Pourcel et al., 2020). The distribution of CRISPR-Cas systems in bacterial phyla is found to have differential representation across the taxonomic levels, where some groups and species are nearly devoid (< 1%) of CRISPR-Cas systems while some carry in almost all (> 95%) genomes (Burstein et al., 2016; Shehreen et al., 2019). The biological significance and basis of the irregular phyletic distribution of CRISPR–Cas systems are not yet proved.

**FIGURE 1:**
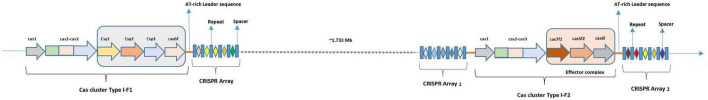
Diagrammatic representation of locus structure of CRISPR-Cas in isolates with co-existence of type I-F1 and type I-F2 systems.

CRISPR-Cas systems have been classified into two Classes (Class 1 and Class 2) and six types (Type I–VI) (Koonin et al., 2017; Makarova et al., 2020). Class 1 includes types I, III, and IV CRISPR-Cas systems, where type I systems are the most prevalent (∼60%) in the bacterial population (Burstein et al., 2017; Hidalgo-Cantabrana et al., 2019). Contrary to Class 2, the Class 1 type I CRISPR-Cas system depends on a multi-subunit CRISPR-associated complex for antiviral defense (Cascade), which further employs Cas3 to degrade the foreign DNA (Westra et al., 2012). Based on the signature gene type I, CRISPR-Cas systems were further divided into seven subtypes (I–A to I–G) (Makarova et al., 2020). Being present in the clinically important (i.e., *Pseudomonas aeruginosa, A. baumannii*) and model bacterial species (i.e., *E. coli*), type I-F CRISPR-Cas is one of the most extensively studied systems. Because type I-F CRISPR-Cas system was first discovered in *Yersinia pestis* (Haft et al., 2005; Cady et al., 2011), its Cascade components were also named Csy (CRISPR subtype Ypest). An updated classification of type I-F CRISPR-Cas system defines type I-F1 loci consisting of four genes: *csy1* (*cas8f1*), *csy2* (*cas5f1*), *csy3* (*cas7f1*), and *csy4* (*cas6f*) while type I-F2 derived from type I-F1 consists of only three genes: *cas5fv* (*cas5f2*), *cas6f*, and *cas7fv* (*cas7f2*), along with the universal adaptation modules (*cas1* and *cas2-3* gene) (Figure 1). In addition, type I-F3, the minimal variant of type I-F1, is associated with Tn7-like transposons, has *cas8f3*/*cas5f3* fused, and lacks *cas1* and *cas2-3* genes (Makarova et al., 2020). Generally, only a single CRISPR-Cas system is present in the majority of the bacterial isolates. However, the co-existence of different types and subtypes was also reported in the single bacterial cell (Carte et al., 2014; Majumdar et al., 2015; Pinilla-Redondo et al., 2020). Type I-F was positively associated with the type IV-A1/2 CRISPR-Cas system and is proposed to compensate for the absence of adaptation modules in type IV (Pinilla-Redondo et al., 2020). Divisive evidence regarding the functional significance of co-occurrence of distinct CRISPR-Cas loci indicates that it can function either independently (Carte et al., 2014) or share components of the process (Deng et al., 2013).

Along with phages, the CRISPR-Cas system can restrict horizontal gene transfer (HGT) occurring through mobile genetic elements (MGEs), thereby limiting the acquisition of potentially beneficial antibiotic-resistant genes (ARGs) (Bikard et al., 2012; Jiang et al., 2013; O’Meara and Nunney, 2019). However, during strong antibiotic selection pressure, the bacterial population often involves loss or inactivation of the CRISPR-Cas system (Jiang et al., 2013; Watson et al., 2018). As the CRISPR-Cas system is present on MGEs (Koonin and Makarova, 2017), it can be attained back through HGT and generalized transduction (García-Martínez et al., 2018; Watson et al., 2018). However, there are some species where the CRISPR-Cas system can co-exist with resistance determinants indicating that a simple rule of selection pressure cannot be universal (Shehreen et al., 2019). The trade-off between retention of CRISPR-Cas system and HGT of beneficial MGEs is being explored to design novel treatments (Lin et al., 2017; Pursey et al., 2018; Pires et al., 2020). Being the natural predators of bacteria, phages can bypass their immune system through anti-CRISPR (ACR) genes. ACR inactivates the CRISPR-Cas system and can facilitate the successful integration of phage into the bacterial genome (Pawluk et al., 2018; Zhu et al., 2018), thereby making them an ideal tool to be used against antibiotic-resistant bacterial communities.

Genome-wide association studies with resistance, virulence, and other genomic determinants are proved to be beneficial to understand the behavior of CRISPR-Cas systems. This study involves the *in silico* analysis of a large set of genome data (*n* = 4,977) available in the public domain to reveal the distribution of CRISPR-Cas in *A. baumannii* and to validate the hypothesis that the presence of co-existing CRISPR-Cas systems may confer a fitness advantage in *A. baumannii* by impacting the dynamics of HGT.

## Materials and methods

### Genomic data

A total of 4,977 genome sequences of *A. baumannii* were downloaded from the NCBI RefSeq database as per availability on 18 January 2021. These genomes are either complete or assembled to different levels; complete genome (245) (containing 535 complete plasmid sequences), chromosome (23), scaffold (1,690), or contig (3,019). These sequences may also contain contigs or scaffolds from episomes. NCBI Annotation (Li et al., 2021) file and genome metadata (genome size, isolation source, host, host disease, submitter, geographic location of the sample) was collected for each isolate (Supplementary Table 1). Guanine-cytosine (GC) percentage was calculated using the pearl script available on git hub.^1^ Multi-locus sequence typing (MLST) was performed using Pasture PubMLST typing scheme^2^ (Jolley and Maiden, 2010).

### Prediction of clustered regularly interspaced short palindromic repeats (CRISPR) arrays, Cas, anti- clustered regularly interspaced short palindromic repeats genes, and phages

The presence of CRISPR array/s was determined using a standalone command-line version of CRISPRCasFinder (v4.2.20), CRISPRDetect (v2.4), and CRISPR Recognition Tool (CRT) (v1.2) (Bland et al., 2007; Biswas et al., 2016; Couvin et al., 2018). CRISPRCasFinder, NCBI-BLASTn (v2.6.0), and NCBI RefSeq genome annotation GFF files were used to determine the presence of *cas* genes (McGinnis and Madden, 2004) and were classified according to the recent classification (Makarova et al., 2011, 2015, 2020). Final interpretations were made based on the same value given by any two out of the three software used. Manual interpretations were made wherever necessary (Supplementary Table 1). Anti-CRISPR (ACR) genes were identified by screening genomes against type I-F anti-CRISPRdb (Dong et al., 2018) using NCBI-tBLASTn with stringent cut-off to avoid false positives, that is, *e*-value ≤ 10^–10^ and bit score ≥ 200 (Shehreen et al., 2019). Integrated phages were discovered by ProphET, a phage estimation tool (Reis-Cunha et al., 2019).

### Phylogenetic tree construction and annotation

The RealPhy (v1.13), a reference alignment-based phylogeny builder (Bertels et al., 2014) was used to construct a phylogenetic tree using whole-genome data of CRISPR-Cas (+) isolates. It directly maps short reads to a reference sequence. It extracts the single nucleotide polymorphisms (SNPs) to infer the phylogenetic tree using the maximum likelihood method for the aligned SNPs positions. We have used the merge option that combines alignments from mapping to multiple reference sequences to remove bias raised due to the alignment to a single reference genome. To visualize and annotate the phylogenetic tree, Interactive Tree Of Life (iTOL)^3^ was used. The phylogenetic tree was uploaded as a Newick file and annotated using tools available on the iTOL website. Data of CRISPR-Cas systems, number of spacers, and MLST groups were overlaid on the phylogenetic tree as multi-value bars and color strips, respectively (Letunic and Bork, 2021). Genome alignment for variability visualization was performed using Mauve v2.4.0 (Darling et al., 2004).

### Prediction of spacer target

A unique non-redundant spacer set for each class was obtained by clustering spacer sequences identified by CRISPRCasFinder with an array-quality score ≥ 4.0, using CD-HIT-EST (v4.8.1) (Huang et al., 2010) with an identity-cutoff of 0.95. Spacers with Ns were removed. NCBI-BLASTn (v2.6.0) was used to predict spacer targets against phage genomes, integrative conjugative elements (ICEs), plasmids, resistance genes, and virulence genes. BLASTn hits with at least 95% sequence identity and coverage were accepted as valid targets (Shehreen et al., 2019). Representative sequences of 99 phages that show interaction with *A. baumannii* were downloaded from the Microbe Vs. Phage (MVP) database (Gao et al., 2018). The life cycle (i.e., temperate, virulent) for these phages was predicted with PHAGEAI (Tynecki et al., 2020). ICEs sequences were downloaded from ICEberg 2.0 database (Liu M. et al., 2019). Plasmid sequences were downloaded from a curated database of plasmid sequences containing 10,892 complete plasmids (Brooks et al., 2019). Acquired resistance gene sequences were downloaded from the ResFinder database of acquired antimicrobial resistance genes (Zankari et al., 2012). Virulence genes were downloaded from the Virulence Factor Database (Chen et al., 2005; Liu B. et al., 2019).

### Prediction of antibiotic resistance and virulence genes

ResFinder (v3.0), with a default minimum threshold and coverage of 0.9 and 0.6, respectively, were used to identify acquired antibiotic resistance genes (ARGs) (Camacho et al., 2009; Zankari et al., 2012, 2017; Bortolaia et al., 2020). ResFinder identifies ARGs from 15 antibiotic drug classes where a complete gene confers resistance. The virulence gene database was obtained from Virulence Finder Database (VFDB) for *Acinetobacter* spp. (Chen et al., 2005; Liu B. et al., 2019).

### Statistical analysis

The presence of the CRISPR-Cas system, virulence, and ARGs are coded as binary variables for each genome. An unpaired two-tailed *t*-test (using GraphPad Prism v6.0.1) was used to determine the association of CRISPR-Cas systems with genome size, number of spacers, and phages. One-tailed paired *t*-test was used to compare the differences between CRISPR-Cas (–) and CRISPR-Cas (+) genomes of the same ST type. Chi-square (χ^2^) test was used to determine the association of the virulence and ARGs with the CRISPR-Cas systems as described earlier (Shehreen et al., 2019).

## Results and discussion

### Clustered regularly interspaced short palindromic repeats (CRISPR)-Cas system in *Acinetobacter baumannii*

**FIGURE 2:**
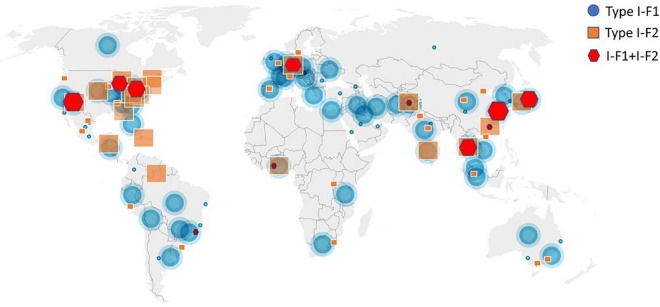
Geographical distribution of CRISPR-Cas (+) isolates across the world. Symbols are merged where two or more isolates coincide.

**FIGURE 3:**
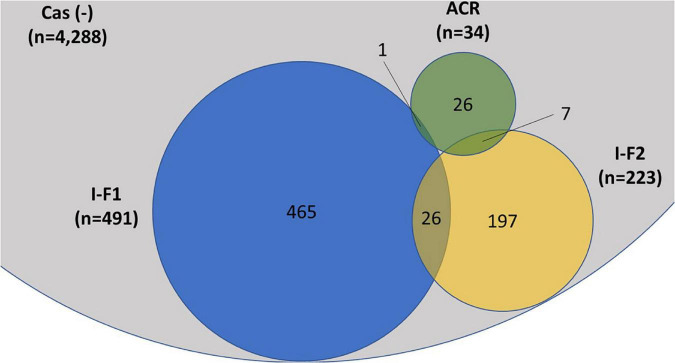
Distribution of CRISPR-Cas type I-F1, type I-F2, and anti-CRISPR (ACR) in the complete (*n* = 4,977) set of *A. baumannii* genomes. A single isolate was found to carry a type III-B system (data not shown). The proportions of degenerated systems (i.e., having either CRISPR or Cas cluster gene/s) are provided in Supplementary Table 2.

A total of 4,977 *A. baumannii* genomes from the NCBI Refseq database were analyzed to determine the frequency and distribution of CRISPR, Cas, and anti-CRISPRs among *A. baumannii*. Based on their source of origin at the time of isolation, genomes were classified into three major categories: clinical (*n* = 4,015), environmental (*n* = 185), and other (in case of unavailability of data) (*n* = 777). Among all, only 13.84% (*n* = 689/4,977) isolates harbored a functional CRISPR-Cas system and were distributed across the globe (Figure 2). These results are in concordance with previous studies that showed the presence of CRISPR-Cas systems in ∼14% of *A. baumannii* isolates (Shehreen et al., 2019; Pursey et al., 2022). Genomes with CRISPR-Cas systems were further analyzed and classified into three categories based on their type: 67.48% (465/689) CRISPR-Cas positive (+) genomes carried only type I-F1, 28.59% (197/689) carried only type I-F2, and 3.7% (26/689) were found to have co-existence of both type I-F1 and type I-F2 (herein after referred as type I-F1 + F2) (Figure 3). Although co-localization of different types of CRISPR-Cas systems is common in bacteria (Carte et al., 2014; Majumdar et al., 2015; Pinilla-Redondo et al., 2020) and are proposed to cooperate to counteract viral escape (Silas et al., 2017), co-localization of variants of type I-F (i.e., I-F1 and I-F2) is rare and not reported to date. A single isolate (strain MRSN7153, United States) was found to harbor a type III-B CRISPR-Cas system and was excluded from downstream analysis. A very low proportion of ACR genes (0.68%; *n* = 34/4,977) were found in *A. baumannii* (Figure 3). Hence, further correlational studies with ACR genes were not performed. Isolates with environmental niches were categorized into hospital and natural environments categories. As expected, a significantly higher percentage (∼56%) of CRISPR-Cas presence was observed in isolates with natural environmental niche compared to clinical (∼15%) and hospital environment niche (∼9%) (Supplementary Table 2), indicating that CRISPR-Cas-mediated immunity provides a clear advantage during defense against phages (Barrangou et al., 2007).

### Organization of type I-F1 + F2 locus

Recent classification based on the multiparametric analysis describes type I loci with *cas3* as a signature gene and type I-F with fused *cas3* and *cas2* genes (Makarova et al., 2011, 2015, 2020; Koonin et al., 2017). On exploring the genomes with type I-F1 + F2 Cas gene clusters, we found that both the systems follow the same organization and features of CRISPR-Cas type I-F1 and I-F2 systems as visualized individually and are distantly (∼1.733 Mb) located in the genome (Figure 1). Interestingly, type I-F2 systems are associated with two CRISPR arrays where one is very well-adapted and spacer rich, compared to the other. In contrast, type I-F1 is only associated with a single CRISPR array. The average spacer size for arrays associated with both type I-F1 and F2 systems is ∼29 bp and belongs to the medium spacers (28–32 bp) category (Pourcel et al., 2020).

### Degenerated clustered regularly interspaced short palindromic repeats (CRISPR)-Cas systems

Under substantial antibiotic exposure, bacterial cells often suppress the function of the CRISPR-Cas system either by partial or complete loss of the CRISPR or Cas genes resulting in a degenerate system (Jiang et al., 2013). We found that 1.49% (74/4,977) isolates exhibited degenerated CRISPR-Cas systems, lacking either CRISPR (*n* = 15) or the complete set of Cas genes (*n* = 59) (Supplementary Table 2). We analyzed 545 plasmids from complete-level genome assemblies for the presence of the CRISPR-Cas systems and found that only 0.18% (1/545) plasmids carried a valid CRISPR array. This accounts for a very low proportion as compared to the average prevalence of 3.4% (546/15,938) across sequenced bacterial plasmids encoding the CRISPR-Cas system (Pourcel et al., 2020). None of the Cas cluster genes were found on the plasmid in *A. baumannii*.

A very low proportion of degenerated CRISPR-Cas systems and plasmids carrying CRISPR arrays suggest that these phenomena are rare but may occur in *A. baumannii*. However, a comparatively very low proportion of clinical isolates (∼15%) harboring CRISPR-Cas compared to environmental isolates (∼56%) suggests that antibiotics may exert selection pressure to lose out or selectively propagate isolates without CRISPR-Cas. Nevertheless, one can infer that the high prevalence of CRISPR-Cas among environmental isolates may be due to the abundance of phages in the environment and not the absence of antibiotics in the case of *A. baumannii*.

### Association of sequence type and clustered regularly interspaced short palindromic repeats (CRISPR)-Cas

**FIGURE 4:**
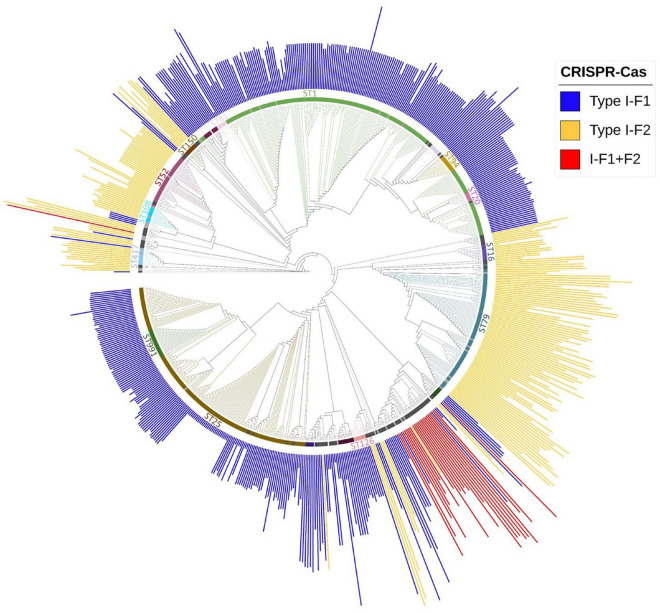
Phylogenetic distribution of different categories of CRISPR-Cas (+) isolates found in *A. baumannii* (*n* = 688). Major STs groups were labeled and are depicted with different colors, while lineages with ≤ 2 and ≤ 5 number of isolates with identical STs were colored with light gray and black, respectively. The height of the bars is proportionate to the total number of identified spacers in CRISPR array/s belonging to that isolate.

Multi-locus sequence typing relies on comparing the sequences of evolutionary conserved but polymorphism-harboring genes (Jolley and Maiden, 2010) and can be employed to compare the phylogenetic diversity among bacterial isolates (van Belkum et al., 2015). Sequence type of all *A. baumannii* isolates was determined using whole-genome sequence following the Pasture scheme and 4,841 isolates belonging to 314 different STs were found, 136 isolates did not belong to any defined ST. We observed that 60.63% (*n* = 3,018/4,977) isolates belonged to ST2 and were devoid of CRISPR-Cas except for 1 isolate which showed type I-F1. Analyzing the distribution of three classes of CRISPR-Cas system among STs, we observed that each class (type I-F1 or type I-F2) predominates within any particular ST with ST138 as an exception which showed the equivalent occurrence of both classes (Supplementary Table 3). Class I-F1 type was entirely observed in isolates with ST1 (*n* = 176/216), ST25 (*n* = 134/140), ST991 (*n* = 18/18), and ST20 (*n* = 9/10). Class I-F2 type was entirely observed in isolates with ST79 (*n* = 77/141), ST52 (*n* = 33/33), and ST16 (*n* = 10/25). However, type I-F1 + F2 were distributed in low frequencies across 12 different ST types, thereby not showing association toward any particular ST (Figure 4).

### Relationship between genome size, phage, and clustered regularly interspaced short palindromic repeats (CRISPR)-Cas

**FIGURE 5:**
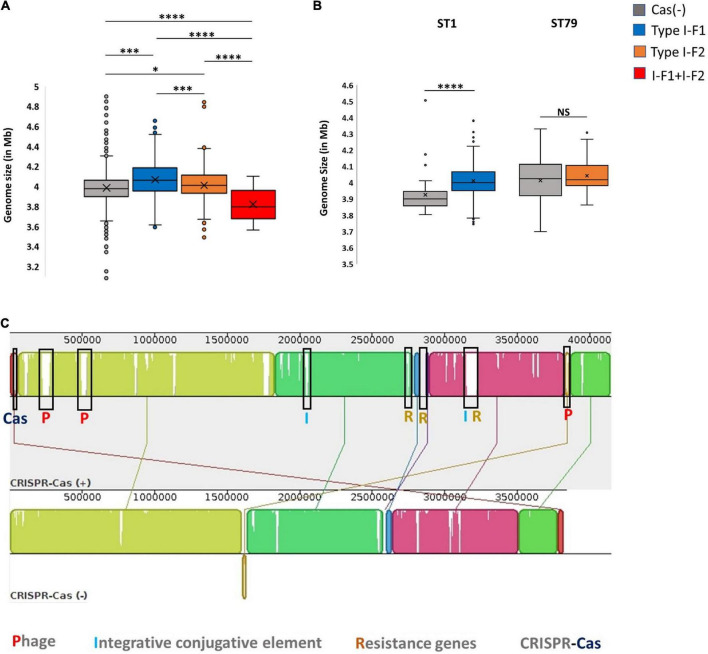
CRISPR-Cas and genome size. **(A)** Genome size comparison of CRISPR-Cas (–) and different categories of CRISPR-Cas (+) isolates. **(B)** Intra-ST analysis of genome size among CRISPR-Cas (–) and (+) isolates in ST1 and ST79. **(C)** Mauve alignment of CRISPR-Cas (+) and (–) ST1 isolates with extreme genome sizes. Where *(*p* < 0.05), ***(*p* < 0.001), ****(*p* < 0.0001) and NS (Not Significant).

The presence of an active CRISPR-Cas system constrains the HGT and phage genome integration, which can limit genome expansion in bacteria and may result in comparatively smaller genome size (Ochman et al., 2000; Lerat et al., 2005; van Belkum et al., 2015; Wheatley and MacLean, 2021; Pursey et al., 2022). Nevertheless, we found that CRISPR-Cas (+) *A. baumannii* isolates were ∼48,982 ± 34,462 bp lengthier than CRISPR-Cas (–) isolates. These results are consistent with the previous study on *A. baumannii* (Pursey et al., 2022). However, categorical observations differentiate skewed data among different types of CRISPR-Cas systems. The isolates harboring only type I-F1 and only type I-F2 CRISPR-Cas systems have unusually larger genomes ∼71,461 ± 29,915 bp (*p* < 0.0001) and ∼21,621 ± 252,021 bp (*p* < 0.0115) than CRISPR-Cas (–) isolates, respectively. However, the genome size of isolates having a type I-F1 + F2 system was smallest (∼1,45,733 ± 23,750 bp smaller (*p* < 0.0001) in comparison with Cas (–) isolates) as compared to other classes (Figure 5A). Intra-ST analysis among prominent ST types harboring specific types of CRISPR-Cas systems confirmed the same trend (Figure 5B). The similarity in different isolates within an ST enables the identification of differential genomic determinants with relatively lower possibilities of discordant variables causing indeterminate effects of CRISPR-Cas systems (Wheatley and MacLean, 2021). Genome alignment of CRSIPR-Cas (+) and (–) isolates belonging to ST1 revealed the presence of phage, ICEs, and ARGs as the contributing factors for genome expansion, thus indicating the redundant function of CRISPR-Cas type I-F1 system (Figure 5C).

Because the active CRISPR-Cas system restricts phages, the observed genome size data were correlated with the integrated phage genome. Average number of phages incorporated (average phage genome size) in each category, Cas (–), type I-F1, type I-F2, and type I-F1 + F2 are 3.17 ± 1.55 (41,613 ± 26,878 bp), 3.91 ± 1.88 (36,789 ± 33,141 bp), 3.91 ± 2.27 (44,580 ± 42,515 bp), and 2.80769 ± 2.1357 (27,823 ± 15,807 bp), respectively. The mean number of integrated phages in CRISPR-Cas (+) isolates of both ST1 (type I-F1) and ST79 (type I-F2) was significantly higher than CRISPR-Cas (–) isolates (type I-F1 –4.73 + 1.86 vs. 3.92 + 0.98; *p* = 0.005; type I-F2– 5.04 ± 1.88 and 3.87 ± 2.54; *p* = 0.000288). Intra-ST comparative analysis of type I-F1 + F2 in CRISPR-Cas (+) and CRISPR-Cas (–) was not performed due to the limited data set available for the associated ST types.

**FIGURE 6:**
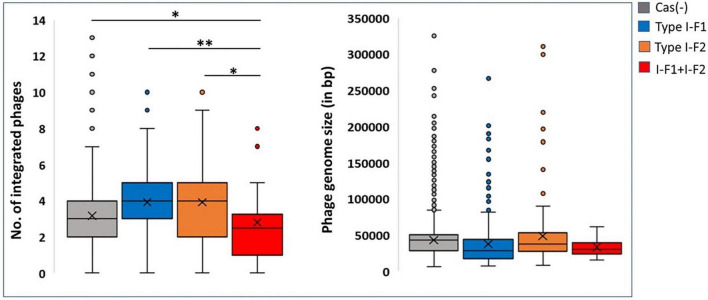
CRISPR and phages: Number of incorporated phages in genomes **(left side)** and the total genome size of the incorporated phage **(right side)**. Where *(*p* < 0.05) and **(*p* < 0.01).

Although a clear decline in the average number of phages in isolates with type I-F1 + F2 was found (*p* = 0.0041), to confirm the activity of the CRISPR-Cas system in restricting the incorporation of phage sequences, we also substantiated the results with phage genome size incorporated in each class, which demarcated a reduction in the size of the phage genome incorporated into the *A. baumannii* isolates with type I-F1 + F2 (Figure 6). The unusually high genome size and integrated phage genomes in isolates with either CRISPR-Cas type I-F1 or I-F2 compared with Cas (-) isolates needs further in-depth studies. We did not find any significant difference in the number of integrated phages in type I-F1 or type I-F2; indeed, their co-existence was more efficient in limiting phage entry, as evidenced by a significantly low number of integrated phages. However, co-occurrence may be associated with the synergistic/additive activity and improving the CRISPR-Cas system’s efficacy but requires more deep, comprehensive, and experimental support.

### Association with resistance and virulence genes

Antimicrobial resistance and virulence are important bacterial traits that help survive and infect the host. Bacteria develop antibiotic resistance either by acquiring resistance genes or through mutations in their genome. It is believed that the CRISPR-Cas system inhibits the acquisition of resistance genes but does not affect the emergence of mutations mediating antibiotic resistance. We investigated the impact of CRISPR-Cas affecting the acquisition of virulence genes and ARGs. In line with previous studies (Shehreen et al., 2019), our extensive analysis did not find an association of CRISPR-Cas (+) isolates with any particular antibiotic class (log frequency ratios ranged from –0.2 to + 0.2) (Supplementary Figure 1A). This suggests that CRISPR-Cas systems do not hinder the dissemination of resistance genes in *A. baumannii*. Similarly, we did not find an association of virulence genes with CRISPR-Cas (+) isolates. However, on analyzing the virulence gene frequencies among three CRISPR-Cas types, we found a strong negative association among isolates with type I-F1 + F2 for biofilm-associated protein (bap) and quorum-sensing genes, *abaI* and *abaR*, with a log frequency ratio of –0.8826, –1.0828, and –1.3767, respectively (Supplementary Figure 1B). The complete gene pool and their respective frequencies for each class of antibiotics and virulence genes found in *A. baumannii* are listed in Supplementary Table 4.

Overall, these results suggest that CRISPR-Cas in *A. baumannii* is not associated with limiting resistance and virulence gene uptake except among type I-F1 + F2 isolates for *bap* and quorum-sensing genes (*abaI* and *abaR*). Our results are consistent with previous studies showing a negative association among CRISPR-Cas (+) isolates for these genes with some modalities (Mangas et al., 2019; Leungtongkam et al., 2020; Tyumentseva et al., 2021).

### What are clustered regularly interspaced short palindromic repeats (CRISPR)-Cas loci spacers targeting?

**FIGURE 7:**
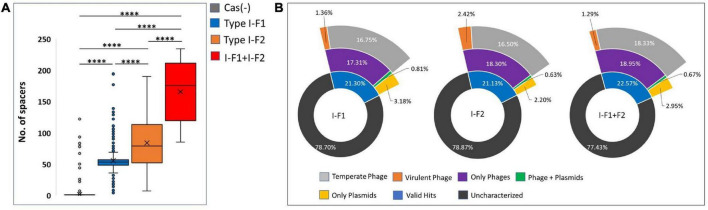
**(A)** Number of spacers incorporated in CRISPR array/s. **(B)** The predicted targets of unique spacers in CRISPR-Cas (+) genomes across different categories of isolates harboring CRISPR-Cas systems. Where ****(*p* < 0.0001).

CRISPR arrays consist of repeats and spacers where repeats were driven intrinsically, while spacers are proved to be acquired from bacteriophages and other mobile genetic MGEs that can provide memory-based immunity to the bacterium. We identified and counted incorporated spacers from each CRISPR-Cas (+) isolate within a valid (evidence level 4) CRISPR array/s. We found a significantly high number of mean spacers (*n* = 164.58 ± 46.41) per isolate in type I-F1 + F2 as compared to isolates with type I-F2 (*n* = 82.87 ± 36.14) and type I-F1 (*n* = 54.51 ± 26.27) (Figure 7A). Hyperactivity of type I-F2 CRISPR-Cas (+) isolates than type I-F1 in acquiring spacers may be correlated with the absence of *csy1* gene (in type I-F2) involved in the formation of Csy complex that negatively regulates Cas1/2-3 complex which functions in adaptation of the CRISPR arrays (Rollins et al., 2017). While in isolates with type I-F1 + F2, a synergistic effect could explain the observed higher spacers per isolate.

Spacerome with unique spacers from each category, that is, type I-F1 + F2 (*n* = 1,338), I-F2 (*n* = 2,224), and I-F1 (*n* = 2,094), were clustered. The number of unique spacers was higher in isolates with type I-F1 + F2 (31.26%), compared to type I-F2 (13.6%) and type I-F1 (8.28%), however, the high number of unique spacers in a population of isolates with CRISPR-Cas type I-F1 + F2 could not be correlated with the higher number of unique spacers per isolate and may be due to small number of isolates belonging to different ST types.

The unique spacers from each defined category were assessed for their potential targets, namely, phages, ICEs, plasmids, virulence factors, and resistance genes. We found that only approximately 21.67% of spacers had valid target hits. Also, it was evident that a single spacer can have a target for an element category, that is, either phage or plasmids. Only a few (∼0.70%) spacers were found to target both phage and plasmids (Figure 7B). The highest proportion of spacers was predicted to target phages in each class. The targeted phage type was further classified based on temperate and virulent groups. Temperate phages are the most common targets for spacers in each category. The limited number of spacers targeting the virulent phages could be attributed to their low abundance (*n* = 5/99) (Figure 7B). In-depth analysis revealed that a single spacer could have multiple targets (a range of 1–37 targets for phages). Spacers that have more than one target against phages are comparatively high in isolates with type I-F1 + F2 (64%), followed by type I-F2 (60%) and type I-F1 (56%). No valid hit was found to target ICEs, resistance, or virulence gene against spacers sets. Maximum remaining spacers had no identifiable target and were designated as dark matter, representing an uncharacterized microbial element (Shmakov et al., 2020).

The presence of spacers with multiple targets reflects the effective management of spacers with remarkable plasticity in *A. baumannii*.

## Conclusion

Broad-scale comparisons across the diversity of *A. baumannii* revealed the distribution (Figure 3) and presence of co-existing CRISPR-Cas systems associated with a higher number of spacers, smaller genome size, and reduced number of integrated phages. It is well reported that CRISPR-Cas systems provide bacteria with an edge against phages. However, it can also target MGEs and may be simply a by-product of the system. We did not find any spacer that can directly target resistance or virulence genes, whereas spacers targeting plasmids that can facilitate the horizontal transfer of resistance and virulence-related genes were observed. However, neither negative nor positive association in CRISPR-Cas (+) isolates with resistance and virulence genes were found, with few exceptions for *bap*, *abaI*, and *abaR* only in type I-F1 + F2 isolates. These contrasting results indicate the existence of cryptic mechanisms for regulating spacers that can target plasmids to acquire and maintain resistance and virulence genes without compromising the phage-based memory in *A. baumannii*. *In silico* data analysis suggested that the co-existence of CRISPR-Cas type I-F1 and F2 systems in *A. baumannii* imparts the hyperactivity against phages without affecting the presence of resistance genes that may significantly hinder the potential of phage-based therapies and the trade-off capabilities. Further research regarding novel treatment strategies should be driven considering the co-existence of CRISPR-Cas systems in *A. baumannii.*

## Limitations and future perspectives

The outcomes of this study correspond to the sequenced *A. baumannii* genomes, including scaffold-level assemblies available in the public domain assessed on 18 January 2021, and oversight newly added and un-sequenced *A. baumannii* population. Notably, the data are inclined toward clinical isolates due to under-represented environmental isolates. Our study shows that type I-F1 and I-F2 CRISPR-Cas systems in co-existence are distantly located; however, this distance may vary on incorporating more complete-level genome assemblies in the dataset. Understanding of complex and diverse CRISPR-Cas systems is rapidly evolving; our analysis does not account for unidentified types and subtypes of the CRISPR-Cas system and anti-CRISPR genes originating from phages. Hence the effect of anti-CRISPR genes is underestimated due to unknown anti-CRISPR proteins associated with the type I-F CRISPR-Cas system. Restricted phage entry as evidenced by a significantly low number of integrated phages in isolates with type I-F1 + F2 was determined *in silico* and requires experimental validation. This study identified maximum spacers with unknown targets, which depict the underrepresented or uncharacterized microbial community. Discovering such new elements may change the dynamics of targets corresponding to spacers being incorporated.

## Data availability statement

The original contributions presented in this study are included in the article/Supplementary material, further inquiries can be directed to the corresponding author.

## Author contributions

GY and RS conceived the idea and designed the study. GY collected and analyzed the data and wrote the manuscript draft. RS reviewed and edited the final manuscript. Both authors contributed to the article and approved the submitted version.

## Footnotes

1
https://github.com/rpotozky/GC-Content-Calculator

2
https://pubmlst.org/organisms/acinetobacter-baumannii

3
http://itol.embl.de
