# Supplementary material for: In silico analysis reveals the co-existence of CRISPR-Cas type I-F1 and type I-F2 systems and its association with restricted phage invasion in Acinetobacter baumannii
Source: Front Microbiol. 2022 Aug 17;13:909886. doi: 10.3389/fmicb.2022.909886 (PMC9428484; doi:10.3389/fmicb.2022.909886)
Supplement: Supplementary file 1 [file Image_1.pdf]

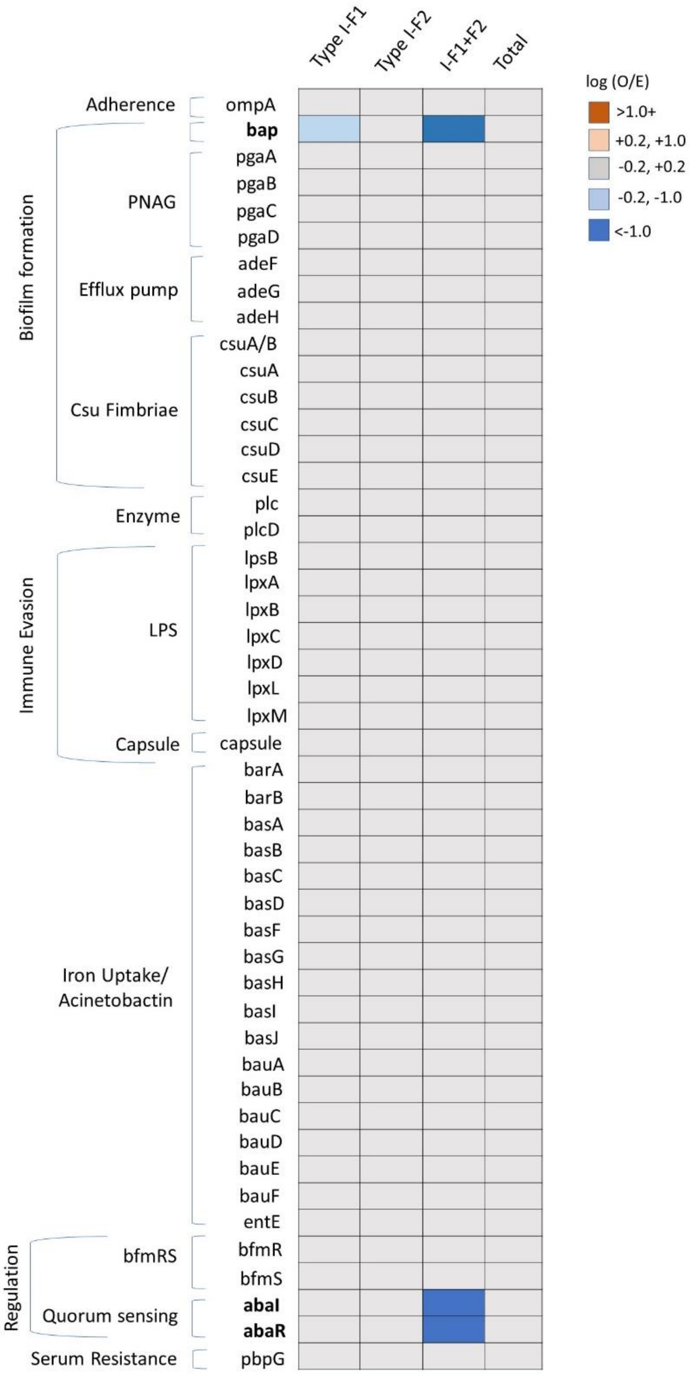

Supplementary Figure 1A. Heat map of log (O/E) frequency ratio, representing association of types of CRISPR-Cas system with virulence factor genes. Where negative log ratio indicates negative association.

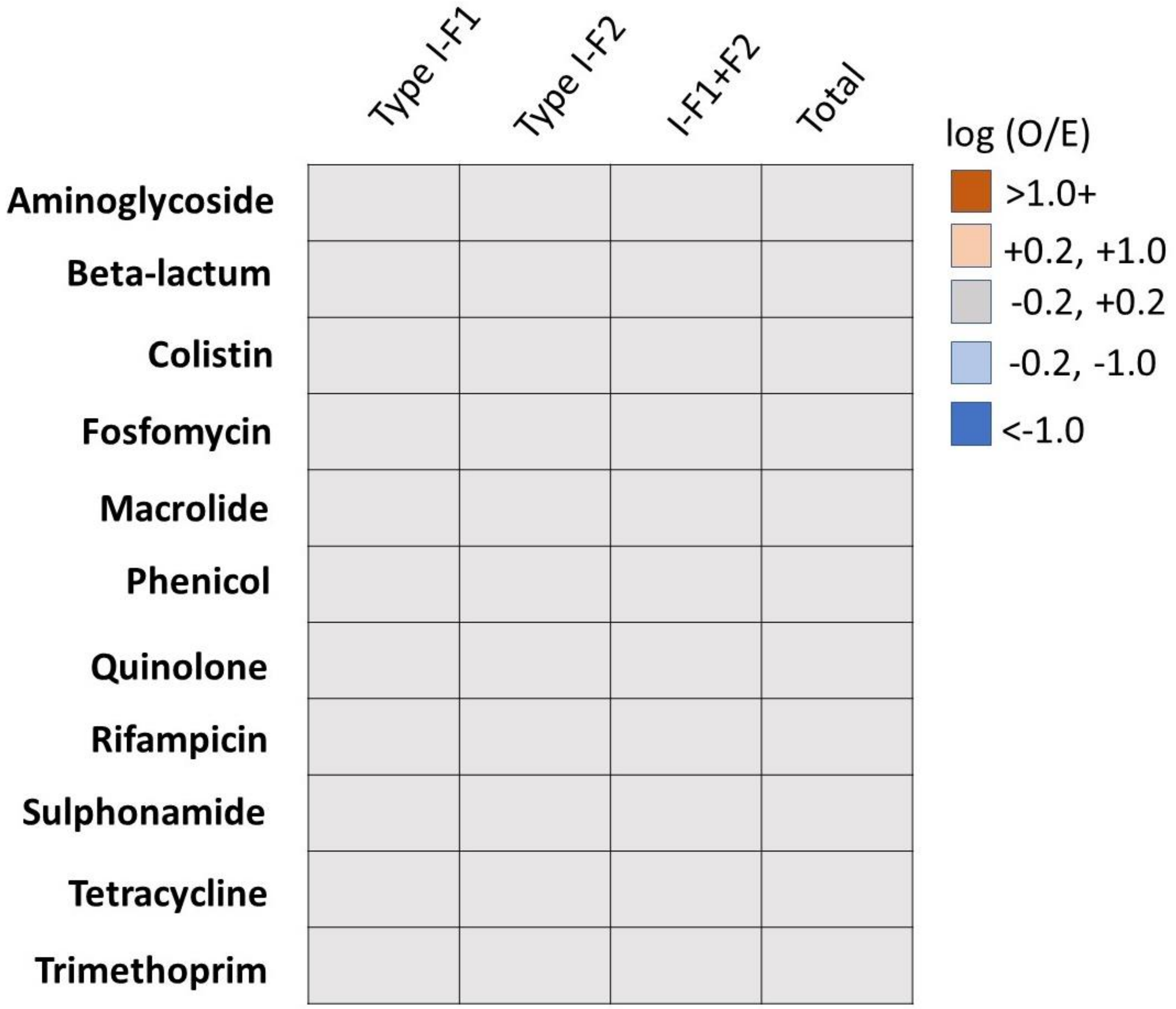

Supplementary Figure 2B. Heat map of log (O/E) frequency ratio, representing association of types of CRISPR-Cas system with resistance genes. Where negative log ratio indicates negative association.
