# Supplementary material for: In silico analysis reveals the co-existence of CRISPR-Cas type I-F1 and type I-F2 systems and its association with restricted phage invasion in Acinetobacter baumannii
Source: Front Microbiol. 2022 Aug 17;13:909886. doi: 10.3389/fmicb.2022.909886 (PMC9428484; doi:10.3389/fmicb.2022.909886)
Supplement: Supplementary file 3 [file Table_2.docx]

**Supplementary Table S2:** Distribution of CRISPR array/s and cas clusters in *A. baumannii* (n=4,977)

|  | **CRISPR (+)** | | | | | **CRISPR (-)** | | | | |
| --- | --- | --- | --- | --- | --- | --- | --- | --- | --- | --- |
|  | Clinical* | Environmental^#^ | | Data not available | Total | Clinical | Environmental | | Data not available | Total |
|  |  | Hospital | Natural |  |  |  | Hospital | Natural |  |  |
| **Cas (+)** | 614 (15.29%) | 8 (8.97%) | 53 (55.82%) | 14 | 689 (13.84%) | 13 (0.32%) | 0  (0.00%) | 1 (0.54%) | 1 | 15 (0.30%) |
| **Cas (-)** | 55 (1.37%) | 0  (0.0%) | 1 (0.54%) | 3 | 59 (1.18%) | 3,333 (83.01%) | 81 (49.09%) | 41  (22.16%) | 759 | 4,214 (84.67%) |

*Total Number of Clinical isolates (n=4,015), ^#^Environmental [Hospital (n=89) and Natural (n=96)] and data not available (n=777).
