# Supplementary material for: In silico analysis reveals the co-existence of CRISPR-Cas type I-F1 and type I-F2 systems and its association with restricted phage invasion in Acinetobacter baumannii
Source: Front Microbiol. 2022 Aug 17;13:909886. doi: 10.3389/fmicb.2022.909886 (PMC9428484; doi:10.3389/fmicb.2022.909886)
Supplement: Supplementary file 5 [file Table_4.docx]

**Supplementary Table 4A:** Virulence Genes found and their respective frequencies.

| Type | Class | Gene | Frequency (presence out of 4,977 isolates) |
| --- | --- | --- | --- |
| Adherence |  | ompA | 4,975 |
| Biofilm formation |  | bap | 4,383 |
|  | PNAG | pgaA | 4,961 |
|  |  | pgaB | 4,943 |
|  |  | pgaC | 4,943 |
|  |  | pgaD | 4,934 |
|  | adeFGH efflux pump | adeF | 4,965 |
|  |  | adeG | 4,972 |
|  |  | adeH | 4,967 |
|  | Csu Fimbriae | csuA/B | 4,333 |
|  |  | csuA | 4,338 |
|  |  | csuB | 4,405 |
|  |  | csuC | 4,441 |
|  |  | csuD | 4,456 |
|  |  | csuE | 4,438 |
| Enzyme | Phospholipase | plcC | 4,921 |
|  |  | plcD | 4,967 |
| Immune Evasion | LPS | lpsB | 4,960 |
|  |  | lpxA | 4,966 |
|  |  | lpxB | 4,965 |
|  |  | lpxC | 4,969 |
|  |  | lpxD | 4,974 |
|  |  | lpxL | 4,961 |
|  |  | lpxM | 4,970 |
|  | Capsule |  | 4,973 |
| Iron Uptake | Acinetobactin | barA | 4,957 |
|  |  | barB | 4,957 |
|  |  | basA | 4,954 |
|  |  | basB | 4,958 |
|  |  | basC | 4,956 |
|  |  | basD | 4,955 |
|  |  | basF | 4,957 |
|  |  | basG | 4,958 |
|  |  | basH | 4,955 |
|  |  | basI | 4,958 |
|  |  | basJ | 4,946 |
|  |  | bauA | 4,955 |
|  |  | bauB | 4,958 |
|  |  | bauC | 4,948 |
|  |  | bauD | 4,956 |
|  |  | bauE | 4,957 |
|  |  | bauF | 4,949 |
|  |  | entE | 4,955 |
| Regulation | bfmRS | bfmR | 4,969 |
|  |  | bfmS | 4,967 |
|  | Quorum sensing | abaI | 4,632 |
|  |  | abaR | 4,557 |
| Serum Resistance |  | pdpG | 4,963 |

**Supplementary Table 4B:** Resistance Genes found and their respective frequencies.

| Antibiotic Class | Gene | Frequency |
| --- | --- | --- |
| Aminoglycoside | aac(3)-I | 1,262 |
|  | aac(3)-Ia | 868 |
|  | aac(3)-IIa | 334 |
|  | aac(3)-IId | 45 |
|  | aac(3)-IV | 2 |
|  | aac(6')-31 | 1 |
|  | aac(6')-33 | 1 |
|  | aac(6')-Iaf | 3 |
|  | aac(6')-Ian | 270 |
|  | aac(6')-Ib | 1,005 |
|  | aac(6')-Ib11 | 1 |
|  | aac(6')-Ib3 | 973 |
|  | aac(6')-Ib-cr | 1,005 |
|  | aac(6')-Ib-Hangzhou | 3 |
|  | aac(6')-Ib-Suzhou | 1 |
|  | aac(6')-IIc | 1 |
|  | aac(6')-Ij | 1 |
|  | aac(6')-Il | 1 |
|  | aac(6')-Ip | 132 |
|  | aac(6')-Ir | 4 |
|  | aacA43 | 2 |
|  | aadA1 | 1,567 |
|  | aadA11 | 3 |
|  | aadA13 | 1 |
|  | aadA16 | 2 |
|  | aadA17 | 1 |
|  | aadA2 | 292 |
|  | aadA24 | 5 |
|  | aadA2b | 27 |
|  | aadA3 | 2 |
|  | aadA5 | 14 |
|  | ant(2'')-Ia | 579 |
|  | ant(3'')-Ia | 6 |
|  | aph(3')-Ia | 1,957 |
|  | aph(3'')-Ib | 2,872 |
|  | aph(3')-IIa | 8 |
|  | aph(3')-VI | 1,260 |
|  | aph(3')-VIa | 1,134 |
|  | aph(3')-VIb | 60 |
|  | aph(4)-Ia | 2 |
|  | aph(6)-Id | 2,879 |
|  | armA | 1,828 |
|  | rmtB | 1 |
| Beta-lactum | blaADC-25 | 4,911 |
|  | blaCARB-1 | 32 |
|  | blaCARB-14 | 19 |
|  | blaCARB-16 | 12 |
|  | blaCARB-2 | 28 |
|  | blaCARB-4 | 23 |
|  | blaCARB-49 | 13 |
|  | blaCARB-5 | 12 |
|  | blaCMH-3 | 1 |
|  | blaCMY-30 | 5 |
|  | blaCTX-M-124 | 5 |
|  | blaCTX-M-15 | 19 |
|  | blaCTX-M-2 | 3 |
|  | blaCTX-M-32 | 1 |
|  | blaCTX-M-55 | 19 |
|  | blaCTX-M-65 | 1 |
|  | blaCTX-M-90 | 1 |
|  | blaCTX-M-98 | 1 |
|  | blaGES-1 | 33 |
|  | blaGES-11 | 30 |
|  | blaGES-12 | 7 |
|  | blaGES-5 | 3 |
|  | blaGES-9 | 3 |
|  | blaIMP-1 | 6 |
|  | blaIMP-14 | 1 |
|  | blaIMP-16 | 1 |
|  | blaIMP-30 | 1 |
|  | blaIMP-4 | 4 |
|  | blaIMP-42 | 1 |
|  | blaIMP-48 | 1 |
|  | blaIMP-54 | 1 |
|  | blaKPC-2 | 1 |
|  | blaKPC-3 | 1 |
|  | blaNDM-1 | 231 |
|  | blaNDM-16 | 5 |
|  | blaNDM-2 | 1 |
|  | blaOXA-1 | 356 |
|  | blaOXA-10 | 77 |
|  | blaOXA-100 | 28 |
|  | blaOXA-106 | 16 |
|  | blaOXA-107 | 3 |
|  | blaOXA-109 | 28 |
|  | blaOXA-111 | 2 |
|  | blaOXA-115 | 1 |
|  | blaOXA-116 | 24 |
|  | blaOXA-117 | 3 |
|  | blaOXA-120 | 68 |
|  | blaOXA-121 | 9 |
|  | blaOXA-123 | 2 |
|  | blaOXA-124 | 15 |
|  | blaOXA-126 | 3 |
|  | blaOXA-128 | 1 |
|  | blaOXA-130 | 1 |
|  | blaOXA-131 | 13 |
|  | blaOXA-132 | 11 |
|  | blaOXA-143 | 3 |
|  | blaOXA-144 | 10 |
|  | blaOXA-146 | 30 |
|  | blaOXA-148 | 1 |
|  | blaOXA-160 | 6 |
|  | blaOXA-164 | 7 |
|  | blaOXA-165 | 30 |
|  | blaOXA-166 | 12 |
|  | blaOXA-167 | 34 |
|  | blaOXA-168 | 30 |
|  | blaOXA-169 | 18 |
|  | blaOXA-170 | 2 |
|  | blaOXA-171 | 34 |
|  | blaOXA-172 | 43 |
|  | blaOXA-173 | 1 |
|  | blaOXA-174 | 1 |
|  | blaOXA-180 | 7 |
|  | blaOXA-2 | 3,331 |
|  | blaOXA-20 | 59 |
|  | blaOXA-200 | 14 |
|  | blaOXA-201 | 1 |
|  | blaOXA-203 | 26 |
|  | blaOXA-206 | 3 |
|  | blaOXA-207 | 10 |
|  | blaOXA-217 | 6 |
|  | blaOXA-219 | 1 |
|  | blaOXA-223 | 17 |
|  | blaOXA-225 | 32 |
|  | blaOXA-23 | 3,048 |
|  | blaOXA-231 | 3 |
|  | blaOXA-234 | 11 |
|  | blaOXA-235 | 48 |
|  | blaOXA-237 | 33 |
|  | blaOXA-239 | 24 |
|  | blaOXA-24 | 209 |
|  | blaOXA-241 | 4 |
|  | blaOXA-242 | 55 |
|  | blaOXA-25 | 46 |
|  | blaOXA-253 | 1 |
|  | blaOXA-254 | 5 |
|  | blaOXA-255 | 1 |
|  | blaOXA-259 | 37 |
|  | blaOXA-26 | 18 |
|  | blaOXA-260 | 4 |
|  | blaOXA-261 | 3 |
|  | blaOXA-262 | 1 |
|  | blaOXA-263 | 4 |
|  | blaOXA-270 | 2 |
|  | blaOXA-297 | 3 |
|  | blaOXA-298 | 1 |
|  | blaOXA-302 | 1 |
|  | blaOXA-312 | 3 |
|  | blaOXA-314 | 10 |
|  | blaOXA-317 | 7 |
|  | blaOXA-337 | 4 |
|  | blaOXA-338 | 2 |
|  | blaOXA-340 | 1 |
|  | blaOXA-341 | 1 |
|  | blaOXA-343 | 59 |
|  | blaOXA-344 | 2 |
|  | blaOXA-345 | 2 |
|  | blaOXA-35 | 2 |
|  | blaOXA-365 | 3 |
|  | blaOXA-366 | 15 |
|  | blaOXA-371 | 10 |
|  | blaOXA-374 | 5 |
|  | blaOXA-378 | 10 |
|  | blaOXA-381 | 1 |
|  | blaOXA-383 | 3 |
|  | blaOXA-387 | 1 |
|  | blaOXA-398 | 30 |
|  | blaOXA-402 | 16 |
|  | blaOXA-407 | 21 |
|  | blaOXA-408 | 3 |
|  | blaOXA-413 | 1 |
|  | blaOXA-420 | 6 |
|  | blaOXA-421 | 1 |
|  | blaOXA-422 | 21 |
|  | blaOXA-424 | 2 |
|  | blaOXA-425 | 4 |
|  | blaOXA-426 | 2 |
|  | blaOXA-429 | 4 |
|  | blaOXA-430 | 52 |
|  | blaOXA-431 | 7 |
|  | blaOXA-432 | 1 |
|  | blaOXA-435 | 30 |
|  | blaOXA-437 | 6 |
|  | blaOXA-440 | 30 |
|  | blaOXA-441 | 2 |
|  | blaOXA-480 | 2 |
|  | blaOXA-482 | 11 |
|  | blaOXA-483 | 31 |
|  | blaOXA-497 | 1 |
|  | blaOXA-500 | 2 |
|  | blaOXA-508 | 4 |
|  | blaOXA-51 | 52 |
|  | blaOXA-510 | 6 |
|  | blaOXA-528 | 15 |
|  | blaOXA-531 | 19 |
|  | blaOXA-536 | 1 |
|  | blaOXA-545 | 1 |
|  | blaOXA-555 | 3 |
|  | blaOXA-556 | 2 |
|  | blaOXA-558 | 14 |
|  | blaOXA-58 | 78 |
|  | blaOXA-64 | 198 |
|  | blaOXA-65 | 219 |
|  | blaOXA-66 | 2,444 |
|  | blaOXA-67 | 33 |
|  | blaOXA-68 | 89 |
|  | blaOXA-69 | 232 |
|  | blaOXA-70 | 12 |
|  | blaOXA-71 | 62 |
|  | blaOXA-72 | 150 |
|  | blaOXA-73 | 31 |
|  | blaOXA-75 | 1 |
|  | blaOXA-76 | 4 |
|  | blaOXA-78 | 8 |
|  | blaOXA-79 | 4 |
|  | blaOXA-80 | 16 |
|  | blaOXA-82 | 687 |
|  | blaOXA-83 | 31 |
|  | blaOXA-88 | 71 |
|  | blaOXA-89 | 2 |
|  | blaOXA-9 | 260 |
|  | blaOXA-90 | 54 |
|  | blaOXA-91 | 23 |
|  | blaOXA-92 | 3 |
|  | blaOXA-94 | 40 |
|  | blaOXA-95 | 56 |
|  | blaOXA-96 | 1 |
|  | blaOXA-98 | 79 |
|  | blaOXA-99 | 3 |
|  | blaPAO | 1 |
|  | blaPER-1 | 74 |
|  | blaPER-7 | 88 |
|  | blaSHV-1 | 31 |
|  | blaSHV-102 | 1 |
|  | blaSHV-12 | 28 |
|  | blaSHV-129 | 2 |
|  | blaSHV-13 | 2 |
|  | blaSHV-143 | 1 |
|  | blaSHV-155 | 2 |
|  | blaSHV-159 | 1 |
|  | blaSHV-161 | 1 |
|  | blaSHV-165 | 1 |
|  | blaSHV-172 | 2 |
|  | blaSHV-31 | 2 |
|  | blaSHV-33 | 1 |
|  | blaSHV-48 | 1 |
|  | blaSHV-5 | 4 |
|  | blaSHV-67 | 1 |
|  | blaSHV-82 | 1 |
|  | blaTEM-104 | 2 |
|  | blaTEM-106 | 1 |
|  | blaTEM-116 | 7 |
|  | blaTEM-122 | 2 |
|  | blaTEM-126 | 1 |
|  | blaTEM-135 | 2 |
|  | blaTEM-141 | 17 |
|  | blaTEM-160 | 5 |
|  | blaTEM-163 | 1 |
|  | blaTEM-164 | 1 |
|  | blaTEM-176 | 1 |
|  | blaTEM-19 | 5 |
|  | blaTEM-191 | 3 |
|  | blaTEM-198 | 2 |
|  | blaTEM-1A | 37 |
|  | blaTEM-1B | 119 |
|  | blaTEM-1C | 1 |
|  | blaTEM-1D | 1,553 |
|  | blaTEM-2 | 27 |
|  | blaTEM-201 | 1 |
|  | blaTEM-206 | 16 |
|  | blaTEM-207 | 1 |
|  | blaTEM-208 | 1 |
|  | blaTEM-209 | 3 |
|  | blaTEM-210 | 1 |
|  | blaTEM-214 | 16 |
|  | blaTEM-216 | 2 |
|  | blaTEM-217 | 2 |
|  | blaTEM-220 | 1 |
|  | blaTEM-229 | 2 |
|  | blaTEM-230 | 1 |
|  | blaTEM-234 | 2 |
|  | blaTEM-29 | 1 |
|  | blaTEM-30 | 2 |
|  | blaTEM-33 | 1 |
|  | blaTEM-34 | 1 |
|  | blaTEM-55 | 2 |
|  | blaTEM-57 | 2 |
|  | blaTEM-71 | 3 |
|  | blaTEM-84 | 6 |
|  | blaTEM-99 | 1 |
|  | blaTMB-1 | 1 |
|  | blaVEB-1 | 5 |
|  | blaVEB-2 | 1 |
|  | blaVEB-3 | 1 |
|  | blaVEB-7 | 1 |
|  | blaZ | 1 |
| Colistin | Mcr4.3 | 2 |
| Fosfomycin | FosA | 18 |
|  | FosA3 | 16 |
|  | FosA4 | 1 |
| Fusidicacid | No gene found | |
| Macrolide | erm(A) | 1 |
|  | erm(C) | 1 |
|  | mph(A) | 2 |
|  | mph(E) | 2,376 |
|  | msr(E) | 2,375 |
| Nitroimidazole | No gene found | |
| Oxazolidinone | No gene found | |
| Phenicol | catA1 | 103 |
|  | catB3 | 46 |
|  | catB8 | 888 |
|  | cml | 165 |
|  | cmlA1 | 161 |
|  | floR | 112 |
| Quinolone | aac(6')-Ib-cr | 1,005 |
|  | oqxA | 1 |
|  | oqxB | 1 |
|  | qnrB19 | 1 |
|  | qnrS1 | 1 |
| Rifampicin | ARR-2 | 135 |
|  | ARR-3 | 9 |
|  | ARR-6 | 1 |
| Sulphonamide | sul1 | 2,127 |
|  | sul2 | 2,183 |
| Tetracycline | tet(39) | 99 |
|  | tet(A) | 97 |
|  | tet(B) | 2,569 |
|  | tet(C) | 2 |
|  | tet(D) | 1 |
|  | tet(G) | 7 |
|  | tet(H) | 1 |
|  | tet(M) | 1 |
|  | tet(X) | 4 |
|  | tet(X3) | 1 |
| Trimethoprim | dfrA1 | 132 |
|  | dfrA10 | 1 |
|  | dfrA12 | 6 |
|  | dfrA14 | 1 |
|  | dfrA16 | 10 |
|  | dfrA17 | 4 |
|  | dfrA19 | 26 |
|  | dfrA20 | 2 |
|  | dfrA27 | 2 |
|  | dfrA5 | 3 |
|  | dfrA7 | 45 |
|  | dfrB1 | 3 |
| Glycopeptide | No gene found | |
